# Polymer modulators in silicon photonics: review and projections

**DOI:** 10.1515/nanoph-2022-0141

**Published:** 2022-07-15

**Authors:** Iman Taghavi, Maryam Moridsadat, Alexander Tofini, Shaheer Raza, Nicolas A. F. Jaeger, Lukas Chrostowski, Bhavin J. Shastri, Sudip Shekhar

**Affiliations:** Department of Electrical and Computer Engineering, University of British Columbia, 2332 Main Mall V6T 1Z4, Vancouver, BC, Canada; Department of Physics, Engineering Physics & Astronomy, Queen’s University, Stirling Hall, 64 Bader Lane K7L 3N6, Kingston, ON, Canada

**Keywords:** electro-optic modulator, electro-optic polymer, silicon organic hybrid, silicon photonics

## Abstract

Optical modulators are vital for many applications, including telecommunication, data communication, optical computing, and microwave photonic links. A compact modulator with low voltage drive requirement, low power, high speed, and compatibility with CMOS foundry process is highly desirable. Current modulator technologies in Si suffer from trade-offs that constrain their power, performance (speed, drive voltage), and area. The introduction of additional materials to the silicon platform for efficient phase shift promises alternatives to relax those trade-offs. Si-organic-hybrid (SOH) devices demonstrate large modulation bandwidth leveraging the electro-optic (EO) effect and smaller drive voltage or footprint owing to a strong EO coefficient. In this study, we review various SOH modulators and describe their path towards integration to silicon, including their challenges associated with aging and temperature. We also briefly discuss other high-performance modulators such as plasmonic-organic-hybrid (POH), photonic-crystal-assisted SOH, and LiNbO_3_.

## 1 Introduction

Modulators play a key role in various photonic integrated circuits (PICs) for versatile applications, including data transmission [1–3], quantum computing [4, 5], optical computing [6–11], and sensing [12]. Si photonic (SiP)-based PICs are now commercially produced in millions in CMOS Si-on-insulator (SOI) foundries [13]. Leveraging the technological advancement of CMOS SOI manufacturing, PICs built in a SiP platform deliver low-cost in high volume production, an unprecedented degree of integration, and reliable long-term performance.

**Figure 1:**
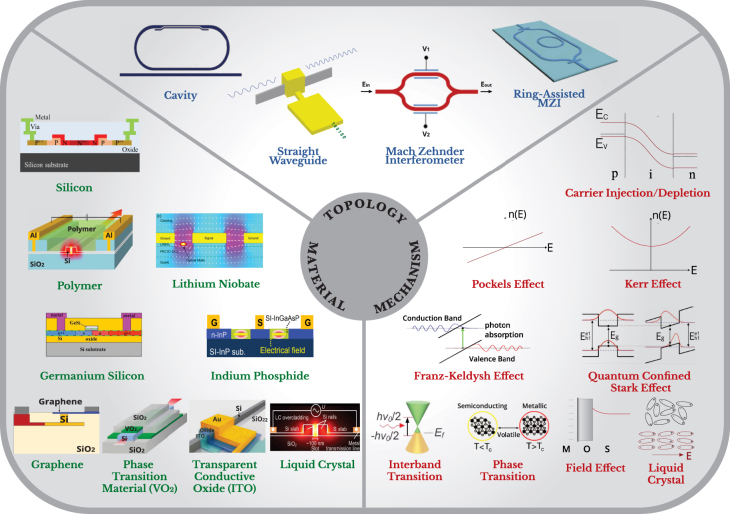
Modulators categorized based on topology, material, and mechanism. Topology can radically change the way an RF signal modulates the light. Different materials make use of different mechanisms for modulation. Not all materials are easy to integrate into a CMOS SOI platform. Some mechanisms are ill-suited for high-speed modulation. Figure reproduced with permission from Optica ([14] [ITO]; [15] [VO_2_]; [16] [InP]; [17] [GeSi]; [18] [Silicon]; [19] [Lithium Niobate]; [20] [Liquid Crystal]).

Several figures of merit (FoMs) are defined to rank modulator performance: modulation efficiency (or equivalently modulation sensitivity or detuning efficiency), insertion loss (IL), electro-optical −3 dB bandwidth (BW), power consumption, footprint, and fabrication complexity. Figure 1 depicts the three main ingredients to realize a modulator including phase or absorption modulation mechanisms, material responsible for the modulation, and the topology based on which a radio frequency (RF) signal modulates the phase or absorption of light.

Si modulators employ the free-carrier plasma dispersion (FCD) effect for high-speed modulation in today’s SiP platform. The Pockels effect is absent in Si, and Kerr and Franz-Keldysh effects are too small in the O/C bands. Thus, FCD, popularly controlled by carrier depletion (in p-n junctions), but occasionally by carrier injection (in p-i-n junctions) or carrier accumulation, is used for Si modulators. When an electric field is applied, *both* the real (Δ*n*) and the imaginary (Δ*α*) part of the refractive index are changed, leading to both electrorefraction and electroabsorption effects, respectively [21]. Thus, pure phase modulation at high speed is difficult to achieve in silicon, unless the slow thermo-optic effect is used. However, the electrorefractive effect is weak, leading to a low modulation efficiency. Consequently, large voltage swings of several volts or a considerable device length of several millimeters are needed. This in turn has created an upper limit of approximately 50 GHz to the BW [22] that can be achieved from a Si modulator with reasonable IL, limited by photon lifetime and RC time constant.

Additional materials can be added to the SiP platform to improve the trade-offs associated with FCD modulation. Germanium (Ge), already integrated into most SiP processes for photodetection, is a natural choice to build electroabsorption modulators (EAM). EAMs rely on Δ*α*. Hence, a straight waveguide can be used for intensity modulation. EAMs employing either the Franz-Kelydysh effect [23] or the quantum confined start effect [24] in GeSi on Si have demonstrated promising performance, with BW exceeding 50 GHz (limited by test equipment) in a CMOS foundry process. Unfortunately, the former is limited to C-band only and the latter, operating in O-band, has a large IL. Since [24] is a recent demonstration, Ge on Si devices are promising for further research and SiP integration. Indium phosphide (InP) is another material that has already been commercialized in a CMOS SOI foundry through a heterogeneous integration approach using plasma-assisted direct bonding of III/V to Si for lasers [25]. InP integration to SiP is important for optical amplification in addition to solving the scaling challenges of PICs [6]. Hence, efforts have been made recently to integrate membrane InP-based devices on a SiP platform to improve modulator efficiency [26].

Other materials can be added to the SiP platform to build electrorefractive modulators relying on Δ*n*, creating phase modulation. A Mach-Zehnder interferometer (MZI) or a resonant cavity must be used for indirect phase to intensity modulation. When Δ*n* is linear with the applied electrical field, the effect is called the Pockels effect. If a nonlinearity is desired, the Kerr effect is usually relied upon. LiNbO_3_ can be added on a SiO_2_ insulator (LNOI) to leverage the Pockels effect and realize low-loss, very high BW modulators, even exceeding 100 GHz [27]. However, their modulation efficiency remains low, and the issue of lithium contamination in a CMOS foundry process must be resolved [28].

Some other materials such as liquid crystal (LC), phase transition material (PTM), phase change material (PCM), and indium tin oxide (ITO) suffer from bandwidth limitations and hence are not attractive for high-speed modulation. Since data transceivers remain the most significant driving force behind SiP technology commercialization, a new modulation technology must achieve better high-speed performance than the existing FCD Si modulators for high-volume production.

This review focuses on organic electro-optic polymers (EOPs) on a SiP platform. Si-organic-hybrid (SOH) modulators leverage the Pockels effect but do not cause contamination challenges in a CMOS foundry. They show excellent overall performance but have challenges in temperature stability in a CMOS foundry integration. While polymer coating and poling can be accomplished as post-processing steps in Si foundries, the additional costs and complexities associated with these extra steps have yet to be surveyed. Poling, an electrothermal process, for all the modulators in a silicon photonic SOI wafer requires applying specific voltages to all such modulators simultaneously while heating the wafer. Since the location of the modulators varies from one design to another on an SOI wafer with various silicon photonic circuits, a scalable solution for such post-processing steps is yet to be demonstrated.

Here, we will first summarize the most critical FoMs of a modulator and compare FCD modulators to their SOH counterparts in Section 2. Section 3 is devoted to the design considerations of the SOH modulators. We also briefly describe other variants of EOP modulators. We also review the recent advances in EOP materials. Section 4 describes the challenges such as aging and temperature reliability, followed by a discussion on the future roadmap.

## 2 Modulator comparison

There are several metrics to be considered when designing a modulator. This section reviews the most important ones to understand the trade-off between them and ways to optimize them.

### 2.1 Topology

SiP modulators utilize phase modulation in various topologies. A Mach-Zehnder modulator (MZM) employs induced phase shift in one or both arms of length *L* to build an intensity modulator. A microring modulator (MRM) utilizes resonance, with the phase shift employed in the cavity. Designed with a quality factor in thousands to tens of thousands, MRMs are compact and can be driven as a capacitive load by CMOS drivers with low power consumption. MZMs are relatively large devices. Often spanning several millimeters in length, they are driven by traveling wave electrodes (TWE) and consume considerable power. However, unlike MZMs, a large quality factor for MRMs makes them highly sensitive to temperature and bias fluctuations. MRMs are also narrow in their optical bandwidth, and not suitable for quadrature modulation. A combination of both topologies, called a ring-assisted MZM, where one or multiple ring resonators are placed in the adjacent arm(s) of an MZM, has been proposed as well [29].

### 2.2 Modulation efficiency

The efficiency of a given modulator (also called detuning factor) at a specific wavelength *λ* is given by *V*_*π*_ *L* = *λ*/2*S*_p_, where *V*_*π*_ is the required voltage to introduce a *π* phase shift, and *L* is the RF electrode length [30]. *S*_p_ is the modulation sensitivity, defined as the effective mode index change versus applied voltage.

**Figure 2:**
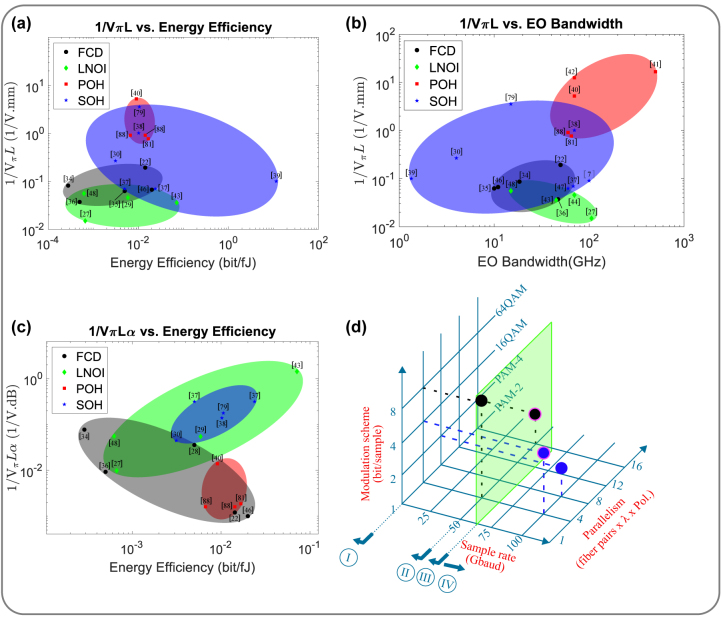
Trade-off between various FoMs for modulators: POH and LNOI modulators, although challenging to integrate to CMOS foundry process, are also shown for the sake of completeness. (a) Inverse of modulation efficiency versus BW. (b) Inverse of modulation efficiency versus energy efficiency, when only dynamic power consumption is taken into account. (c) Inverse of loss efficiency versus energy efficiency, when only dynamic power consumption is taken into account [46–48]. (d) achieving 1.6 Tb/s aggregate data rate by increasing the modulator Gbaud operation, modulation scheme and parallelism (number of fiber pairs × wavelength × polarization). Gbaud is limited to <1 for category I (e.g. LC, thermal, PCM, PTM and ITO), <50 for category II (FCD), <70 for category III (InP and GeSi EAM) but can reach 100 for category IV (LNOI and EOP). The advantages of SOH modulators over FCD are clearly seen. 1.6 Tb/s can be achieved using 50 Gbaud FCD modulators operating in PAM4 with either 16 fibers or 16 *λ*, or in 64QAM dual-polarization (DP) with 2 *λ*. With 100 Gbaud EOP modulators, 1.6 Tb/s can be realized in PAM4 with half the fibers or *λ*s, or in 16QAM DP with 2 *λ*.

It can be shown that for EOP modulators experiencing the Pockels effect,

(1) $$S_{\text{p}}=\frac{\partial n_{\text{eff}}}{\partial V_{\text{in}}}=\frac{1}{2}n^{3}r_{33}\frac{\Gamma }{d}$$

where *n* is the EOP material refractive index inside slot, *r*_33_ is the electro-optic (EO) tensor coefficient of the material, Γ is the field overlap integral between electrical and optical fields [31], and *d* is the separation between the two electrodes through which the modulating signal is applied. To maximize *S*_p_ and thus minimize the *V*_*π*_ *L*, one should engineer both the material (i.e., *n* and *r*_33_) and the device structure (i.e. *d* and Γ). EOPs naturally possess physical and chemical properties that make them ideal candidates to improve the abovementioned parameters. EOPs have a very large EO coefficient (i.e. *r*_33_). Indeed, *r*_33_ values up to 460  pm/V [32] have been reported for cross-linked polymers compared to ∼30  pm/V [33] for their inorganic LiNbO_3_ counterparts. As we will see later, *n* ^3^ *r*_33_, another electro-optic FoM, can reach 3100  pm/V for EOP materials compared to ∼(2.3)^3^ × 30 = 365 pm/V for LiNbO_3_. An interesting characteristic that can add another order of magnitude improvement to the modulation efficiency is the physical state. Polymers are typically liquid when are applied to the device structure. It allows infiltration into nanoscale geometries benefiting from larger Γ due to stronger light–matter interaction and also smaller electrode gaps. This contrasts with other materials (in solid format) such as graphene, PCM, and ITO usually added as a coating to exploit the comparatively much weaker optical field in the cladding. There is no surprise, therefore, that the lowest *V*_*π*_ *L* values (among high-speed modulators) belong to the EOP modulators (see Table 1 and Figure 2a). It means a shorter *L* and hence a smaller footprint for the same *V*_*π*_, or a smaller *V*_*π*_ for the same *L*. Lower swing voltage for a compact modulator presents the opportunity to relax or even eliminate the high-swing driver requirements and lower overall power consumption by connecting the modulator directly to the pre-driver CMOS circuits.

**Table 1:** FoM comparison among the best-reported candidates of various modulation mechanisms, topology, and material. Energy consumption only includes dynamic power, and ignores the effect of static power and IL. POH and LNOI modulators, although challenging to integrate to CMOS foundry process, are also shown for the sake of completeness.

Platform	Topology	EO BW	Propagation loss	Insertion loss	Half wave	Modulation efficiency	Loss-efficiency	Line rate	Energy	Footprint	Ref.
			*α* (dB/mm)	IL (dB)	voltage *V*_*π*_ (V)	*V*_*π*_.*L* (MZM) (V.mm)	*V*_*π*_.*L*.*α*	(Gbps)	consumption	Arm length (MZM)	
						shift (MRM) (pm/V)	(V.dB)		(*f*J/bit)	Ring radius (MRM)	
										(mm)	
FCD	MZM	18	1.5	3.6	11.5	11.5	17.25	25	3400	1	[34]
FCD	MZM	10	1.75	8.7	3.2	16	28	20	200	5	[35]
FCD	MRM	50	160	3.2	260	5.2	832	64	70	0.01	[22]
FCD	MZM	47	4	15	7.7	27	108	40	2000	3.5	[36]
GeSi	Straight WG	>50	110	4.4	2	0.08	8.8	50	13.8	0.04	[23]
GeSi	Straight WG	50	190	7.6	2	0.08	8.8	60		0.04	[24]
SOH	MZM	68	0.22	1.76	1.8	14.4	3.16	200	42	8	[37]
SOH	MZM	70	7.2	8	0.9	0.99	7.2	100	98	1.1	[38]
SOH	MZM	100	4.23	2	22	11	46.53	140	–	0.5	[7]
SOH	MRM	1.34				10			0.087		[39]
POH	MZM	70	375	6	12	0.192	72	72	110	0.016	[40]
POH	MZM	500	500	10	3	0.06	30			0.02	[41]
POH	MZM	>70	544	13.6	3.6	0.09	49	72	–	0.025	[42]
LiNbO_3_	MZM	45	0.025	0.5	1.4	28	0.7	210	14	20	[43]
LiNbO_3_	MZM	108	1.5	7.6	13.4	67	102	150	1500	5	[27]
LiNbO_3_	MZM	70	0.83	2.5	7.3	22	18.3	100	170	3	[44]
LC	MZM	0.001	10	10	0.022	0.022	0.2			1	[45]

A more comprehensive way to formulate modulator efficiency is the loss efficiency *α* × *V*_*π*_ *L* (in V.dB) where *α* is the loss per unit length (in dB/mm). We will see shortly that EOP modulators featuring small *V*_*π*_ *L* are built based on a slot waveguide structure contributing to higher *α*. Nevertheless, Figure 2c and Table 1 reveals that, despite its higher *α*, polymer-based technology can still demonstrate some of the smallest *α* × *V*_*π*_ *L* due to its significantly reduced *V*_*π*_ *L*.

### 2.3 Electro-optical bandwidth

The BW of a modulator depends upon the velocity matching between the RF and optical signals. The BW constraint is given by: (*BW* × *L*)_max_ ≈ 1.9*c*/*π*|*n*_RF_ − *n*_o_|, where *c* is the speed of light in vacuum, *n*_RF_ is the RF effective index of the electrode, *n*_o_ is the optical effective refractive index of the waveguide with EOP, respectively [49]. Unlike LiNbO_3_, *n*_o_ for the EOP waveguide can be optimized with different geometry and material, to match closely with *n*_RF_. Thus, a low dielectric constant dispersion and a small velocity mismatch can lead to large BW for EOP modulators, exceeding 100 GHz [41, 50, 51]. For TWE MZMs, two other factors also constrain the BW: (1) mismatch between the driver impedance, the characteristic impedance of the modulator electrodes and termination impedance, and (2) the RF attenuation. Since EOP MZMs can be made significantly smaller, they can be driven as lumped element devices, permitting large BW [41, 50, 52]. Figure 2b and Table 1 show the BW attained by various EOP modulators, limited mainly by the RC time constant.

Increasing the modulation complexity, from 2-level pulse-amplitude modulation (PAM-2) to PAM-4 or PAM-8 or using quadrature modulation schemes such as QPSK/8QAM/16QAM, can also increase the overall data rate (Gb/s). This of course leads to complex drivers, transimpedance amplifiers (TIAs), analog-to-digital converters (ADCs), digital-to-analog converters (DACs), digital signal processing (DSP), as well as a large inter-symbol interference (ISI) penalty. Putting more lanes in parallel, either in wavelength, fiber or polarization, also increases the aggregate data rate [53]. As Figure 2d shows [54], FCD modulators use these techniques to increase the overall data rate. But a modulator with a larger BW naturally permits a larger data rate (Gigabaud) and hence eases the power, area, and cost concerns. EOP modulators, with BW exceeding 100 GHz, promise an attractive path towards 800 Gb/s and 1.6 Tb/s operations. EOP modulators are also capable of zero-chirp operation even when driven with single-ended drivers [55].

### 2.4 Energy consumption

The total amount of energy that a given modulator consumes depends on several factors - static power consumption, dynamic power consumption, and optical IL.

Static power consumption depends upon the modulator structure and carrier dynamics. MRMs are compact and do not often need termination for impedance matching consuming negligible static power from the drivers. Long MZMs, on the other hand, are implemented as doubly terminated TWEs and consume significant static power. Because of their superior modulation efficiency, EOP MZMs can be made with *L* small enough to be driven as lumped element devices. Their static power can be made significantly smaller compared to FCD MZMs. The carrier dynamics involved in the phase shift mechanism determine the operating current of the modulator. Carrier-depletion FCD modulators and Pockels-based modulators such as LiNbO_3_ and SOH do not consume significant bias current.

Dynamic power at a given frequency (proportional to $CV_{\text{pp}}^{2}$ ) is also smaller in the case of EOPs not only because of the low peak-to-peak voltage swing (*V*_pp_) requirements (from the lower *V*_*π*_) but also due to the reduced capacitance (in non-slotted structures). Joule per bit or bit per Joule is typically used to measure energy efficiency for data communication. For computing, Joule per multiply-accumulate (MAC)) is a common metric. Figure 2b shows a scatter plot of the inverse of modulation efficiency versus energy efficiency (bit/*f*J) for various modulators. Since most of the cited references only provide dynamic power unfortunately, the energy is shown for dynamic power only. Any deductions made from this plot should keep this limitation in mind.

Finally, a higher *α* and a longer *L* leads to a large IL for modulators. Since lasers are very inefficient and typically exhibit a wall-plug efficiency of less than 10%, compact modulators with small IL are highly desirable. EOP modulators remain competitive in this regard too, as seen in Table 1. In Figure 2(c), a scatter plot of the inverse of loss efficiency versus BW is shown.

## 3 EOP modulator design

As discussed earlier, we must consider various device and material aspects for the desired modulator performance. Light–matter interaction and material are the two most prominent factors that must be engineered for a high-performance operation. Fabrication complexity also imposes significant constraints on the available choices.

### 3.1 Light–matter interaction

**Figure 3:**
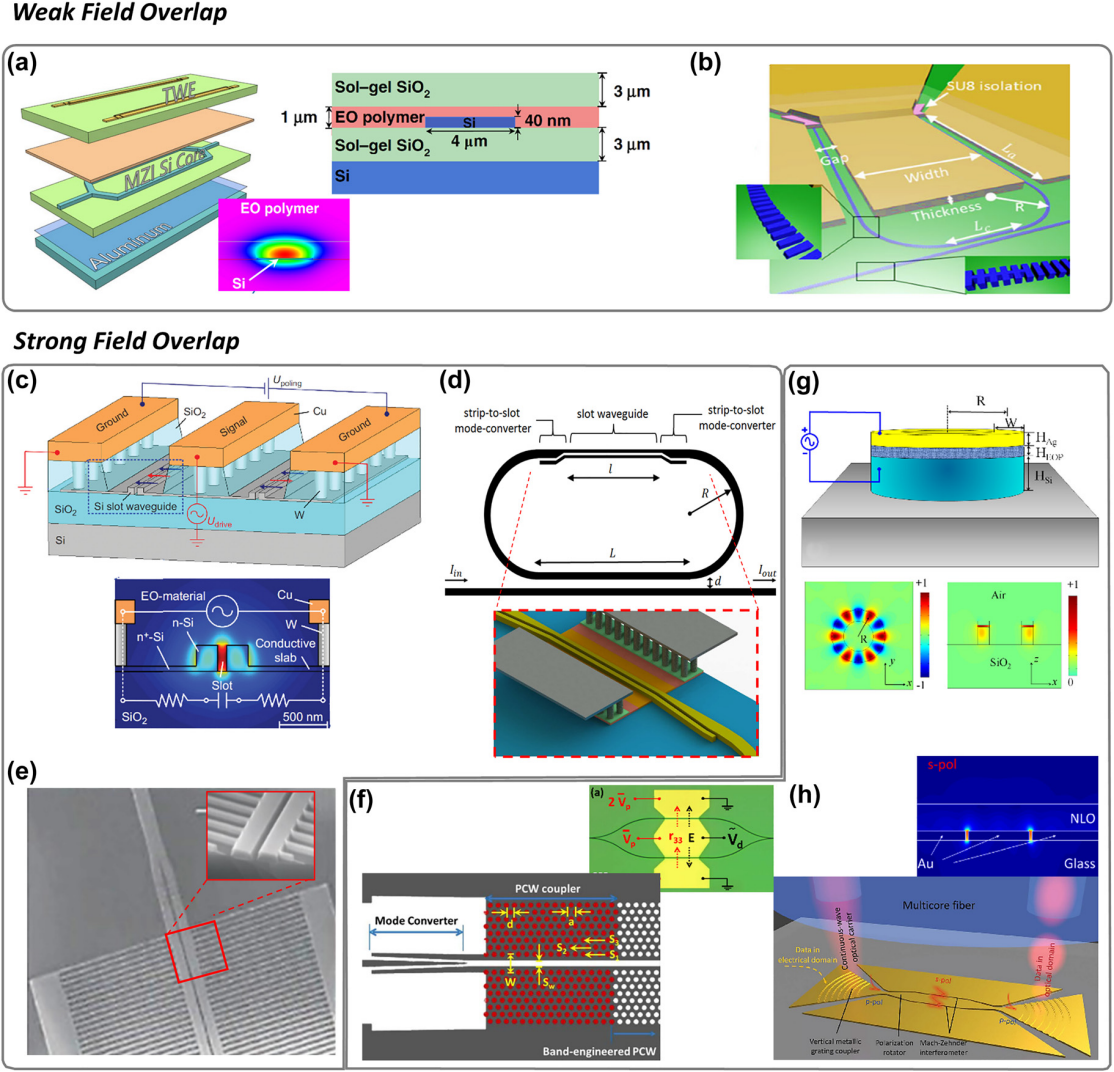
Categorizing various waveguide structures based on light–matter interaction: Weak-field overlap [top]: (a) Non-slotted Si waveguide coated with EOP [37] in an MZM topology. Skinny waveguides (40 nm) increase leakage to improve field overlap. (b) An SWG-assisted version in a racetrack MRM topology [56]. Strong-field overlap [Bottom]: (c) Cross-section of a slot-waveguide MZM. Optical field mode confinement inside the slot and effective overlap with the RF mode field (inset) [50]. (d) Partially slotted waveguide utilized in a race-track MRM featuring a compact strip-to-slot mode converter, high modulation efficiency and quality factor compared to fully slotted MRM [57] (e) Segmented slot waveguides benefit from simpler (i.e. single etch). fabrication due to narrow fins carrying the electric signal for higher speed and lower IL operation [58]. EOP modulators with complicated manufacturing: (f) Photonic crystal (PC)-assisted slot-waveguides featuring enhanced modal field and wider slot width which can alleviate incomplete poling issues [59]. (g) A POH MRM infiltrated with EOP [52]. (h) A POH phase shifter used in an MZM with ultra-low energy consumption and large BW [40]. Figure reproduced with permission from [37], SpringerNature (a); [56], Wiley (b); [50], SpringerNature (c); [59], Optica (f); [52], Optica (g). Figure adapted with permission from [58], AIP (e); [57], SPIE (d); [40]; Science (h). Distributed under Creative Commonslicense CC BY 4.0.

Various types of waveguides can be used to reap the benefits of EOP polymers. From a light–matter interaction perspective, one can categorize all EOP modulators into two main categories: (a) Weak-field overlap: light propagates inside the non-slotted Si (or SiN) waveguide with a slight leakage into the EOP material as the waveguide cladding. (b) Strong-field overlap: light propagates primarily inside the EOP material within the slot(s) of the waveguide. As discussed next, the latter benefits from the larger field overlap between the RF (modulating) and the optical (carrier) fields. Various embodiments of the two categories are illustrated in Figure 3.

#### 3.1.1 Weak-field overlap

Figure 3a shows a non-slotted modulator. Here, a strip waveguide of Si is used as the primary propagation medium coated by EOP. The weak optical field leaking from the waveguide interacts with the EOP. TWEs at the top and the bottom apply the electric field through the SiO_2_ layers. Contact poling is then carried out by heating the EOP to its glass transition temperature (*T*_g_) under this electric field. The chromophore dipoles are thus aligned, and the EOP achieves its *r*_33_. Due to the large separation between the two electrodes, the field overlap is weak, and the *V*_*π*_ *L* product is large. This necessitates long TWEs. However, the large separation keeps the capacitance low, improving the BW. The long TWEs eventually limit the BW. Since the light primarily travels inside the un-doped waveguide, IL remains low. Finally, unlike their counterparts with strong-field overlap, these modulators neither require a doped region nor multiple steps in making the waveguides.

In Figure 3a, sol-gel layers are used to enhance the poling efficiency (maximizing *r*_33_ while avoiding dielectric breakdown). One way to improve the field overlap (and hence lower the *V*_*π*_ *L*) is to use subwavelength grating (SWG)-assisted or Bragg-assisted waveguides [56], as depicted in Figure 3b.

#### 3.1.2 Strong-field overlap

One way to improve the optical and electrical field overlap is to employ a slotted rib waveguide. Figure 3c shows the cross-section of a push-pull MZM with slotted waveguides in both its arms. The relative permittivity of the polymer material in the slot is smaller than that of Si. Therefore, the optical field is intensified inside the slot to maintain a continuity of the displacement field (D) across the slotted waveguide. As a rule of thumb, Γ is typically 10× higher in slotted EOPs compared to their non-slotted counterparts depending on the slot size [4]. Therefore, excellent modulation efficiencies have been demonstrated in the slotted EOP modulators: for instance 0.32 V mm in [60]. Other examples of the best-reported devices are shown in Table 1. Slot waveguide-based modulators require strip-to-slot converters that often introduce significant IL. But recently, a converter featuring a logarithmic design has demonstrated a loss of just 0.02 (±0.02) dB [61], shown in Figure 3d.

Slot widths as low as 75 nm have been reported [62]. However, smaller slot widths could be challenging in fabrication, infiltration, and poling. To facilitate infiltration into small slots, the oxide underneath the slot can be over-etched [63]. TWEs and either vias or air bridges carry DC poling and RF signals to the rails. The RF electric field is applied to the two rails [39, 64] using a partially etched slab layer (also called pedestal). This layer is made of highly doped silicon. A careful design is required for this slab‘s thickness, width and doping levels to avoid: (1) dragging too much modal field to the silicon surrounding the slot, (2) excessive IL, and (3) excessive resistance. This results in a compromise between various metrics of the modulator (BW, IL, *V*_*π*_ *L*). For instance, the thinner the slab layer, the lower the field outside the slot, and the higher the modulation efficiency. But the resistance is also increased, increasing the overall RC time constant. A smaller slot width also leads to increased capacitance. Finally, the structure suffers from high optical loss mainly caused by the sidewall roughness of the slot and, to a lesser extent, due to the lightly doped waveguide. As a result, MRMs built out of the slotted waveguide have a lower quality factor. Stronger field overlap can be utilized in a fully slotted MRM structure [65–67]. The light travelling in either a slotted or non-slotted bus waveguide is coupled to a fully slotted ring resonator. A larger ring radius is typically required to decrease the bending loss due to radiations. A partially slotted MRM has been proposed to sacrifice some of the detuning efficiency for the sake of smaller bending loss [68, 69], as illustrated in Figure 3d. This resulted in a higher quality factor, and therefore, a better modulation efficiency.

A double (or multiple) slot waveguide structure is proposed in [70] to maximize the field enhancement and modulation efficiency. However, the additional IL caused by the slot sidewall roughness can nullify the benefits of this approach. Another alternative is a finger-based structure (fin-based or segmented-based), as shown in Figure 3e [58]. Instead of the slab layer, a set of narrow, doped fingers are employed. The subwavelength segments provide a quasicontinuous electrical contact thus reducing IL and increasing the BW. Only one single etch step is required to fabricate both the slotted waveguide and the fingers, unlike the case of a plain rib slot waveguide. Design considerations must still be considered to avoid excessive IL and resistance associated with the fingers. Various versions of this topology have also been proposed, including a fully suspended, Bragg-assisted slotted waveguide [71]. Improvement in modulation efficiency up to an order of magnitude is theoretically shown in [72].

Polymer modulators, in general, can achieve much better performance, but their integration to CMOS-compatible silicon photonic platforms remains elusive. These include EOP modulators with horizontal slot waveguides fabricated using chemical vapor deposition (CVD) [73–75] and all-polymeric waveguides [76, 77]. With its highly confined optical mode, a 2-D photonic crystal (PC) can be used to assist the slot waveguide and maximize Γ [62, 67, 78, 79]. Based on (1), a larger Γ permits a larger *d* and a relaxed slot fabrication for a given *S*_p_ or *V*_*π*_ *L*. A wider slot width improves the infiltration of the EOP and the extent of poling. Conversely, a higher modulation efficiency can be obtained for the same narrow slot width. Figure 3f shows an MZM with PC-assisted slot waveguides embodied in both arms. Limited optical bandwidth and manufacturing variability associated with 2D PC structures remain two crucial challenges. Finally, the most noteworthy example is a plasmonic-based structure, i.e., a plasmonic-organic-hybrid (POH) [41, 80, 81]. Figure 3g and Figure 3h depict the schematic and cross-section of two such devices infiltrated with EOP. Plasmonic nanostructures utilize surface plasmons to greatly enhance light–matter interaction and hence Γ. Thus, a large phase shift efficiency, shorter length, relaxed slot width, lower capacitance and large bandwidth- all are possible. Since no slab layer is required to carry the RF signal to the EOP-filled slot, the total resistance is also significantly reduced. Consequently, high-speed operation up to 500 GHz with excellent *V*_*π*_ *L* of 0.07 V mm [41] has been reported for this sub-category. The downside of POH is its incompatibility with CMOS-based foundries. Particularly, POH requires the fabrication of metal contacts of the plasmonic structure very close to the silicon tapers. In addition, there should be an opening in the oxide on top of the device to infiltrate EOP into the slot. However, such an opening will expose the metal contacts, and metals are typically not allowed to be exposed in a dielectric plasma etch tool.

### 3.2 EOP material

Numerous nonlinear optical polymers have been developed and advanced over the past three decades [32]. Designing a polymer molecule focuses on optimizing the EO tensor matrix (*r*_33_). The ratio between the average achieved *r*_33_ to the poling field is defined as poling efficiency (*r*_33_/*E*_poling_). It is a better metric for comparing EOP materials since it averages the *r*_33_ data over multiple experiments independent of the poling field. In [82], *r*_33_ as high as ∼1100 pm/V and a poling efficiency of ∼11.6 nm^2^/V^2^ has been reported. It is worth noting that *r*_33_ is a material property and should be differentiated from *r*_33, in-device_ which incorporates *r*_33_ as well as device structure (i.e. light–matter interaction). In other words, a larger *r*_33_ together with a stronger light–matter interaction result in a larger *r*_33, in-device_.

Aside from a large *r*_33_, a small refractive index of the EOP, *n*_EOP_ or briefly *n*, is usually desired to create the maximum contrast between the Si waveguide and EOP material, $n_{\text{Si}}^{2}$ / $n_{\text{EOP}}^{2}$ , particularly in a slot waveguide. This can result in higher optical field confinement inside the slot, and consequently, higher Γ. On the other hand, one can isolate the effects of device structure (Γ and *d*) and EOP material properties ( $n_{\text{EOP}}^{3}$ and *r*_33_) by defining another FoM $\left(n_{\text{EOP}}^{3}r_{33}\right)$ . Based on Eq. (1), larger $n_{\text{EOP}}^{3}r_{33}$ is required to reduce *V*_*π*_ *L*. A summary of some of the EOPs and the associated FoMs can be found in Table 2. A comprehensive study of various EOP materials can be found in [83]. In [84], authors have achieved very high $n_{\text{EOP}}^{3}r_{33}$ by studying various combinations of donor and acceptor molecules to improve poling efficiency and thermal stability. Other parameters such as good solubility and compatibility with the polymer matrix should also be considered.

**Table 2:** Summary of EOP materials.

Material	*r*_33_ bulk	*r*_33_ in-device	*n* refractive	*n* ^3^ *r*_33_ bulk	*n* ^3^ *r*_33_	Measurement	Glass	Poling field	Poling efficiency	Ref.
	(pm/V)	(pm/V)	index	(pm/V)	in-device	wavelength	transition	(V/*μ*m)	(nm^2^/V^2^)	
					(pm/V)	wavelength	temp (°C)		(nm^2^/V^2^)	
SEO100	166	–	–	–	–	1310/1550	140	–	–	[85]
SEO125	125	1190	–	–	–	1310	–	–	–	[86]
PVT	–	80	1.67	–	372	–	172	–	–	[87]
JRD1	–	390	1.81	3850	2300	1550	82	–	–	[60]
PMMA/DR1	12.8	–	–	–	–	–	–	–	0.31	[57]
DLD164	133	150	–	–	–	1550	66	280	0.54	[88]
YLD124	100	30	–	–	–	1550	–	150		[89]
BNA	24		1.77	135	–	1538	105	–	–	[90]
YLD124	230	30	1.70	1129	147	–	105	150	0.23	[30]
PSLD41	90	98	1.72	458	499	–	103	312	0.26	[91]
YL156/PMMA	50	19	–	–	–	1550	>70	49	–	[92]
YL124/APC	120	44	–	–	–	1550	85	45	–	[92]
YLD124/PSLD41	285	230	1.73	1475	1190	1550	97	250	0.92	[92]
AJ309	–	110/142	1.64	–	485/626	1550	135	200–400	–	[77]
M3	18	–	–	–	–	1550	167	–	–	[7]
M1	70	230	–	–	–	1550	–	–	–	[93]
AJ/CKL1	90	735	1.63	390	3183	1550	145	86	–	[63]
AJSP100	65	40	1.54	237	146	1550	103	100	–	[94]
HLD1/HLD2	460	–	1.89		3100	1310	170	1300	2.3	[32]
C1	273	–	2.12	2601	–	1300	117	80–135	–	[84]
YLD124	325–351	–	1.81–1.98	1927–2522	–	1310	94	–	2.3	[95]
	220		1.77–1.91	1219–1532		1550				
BAH13	1100	208	1.85	4700	∼1317	1550	–	∼85	11.6	[82]

**Figure 4:**
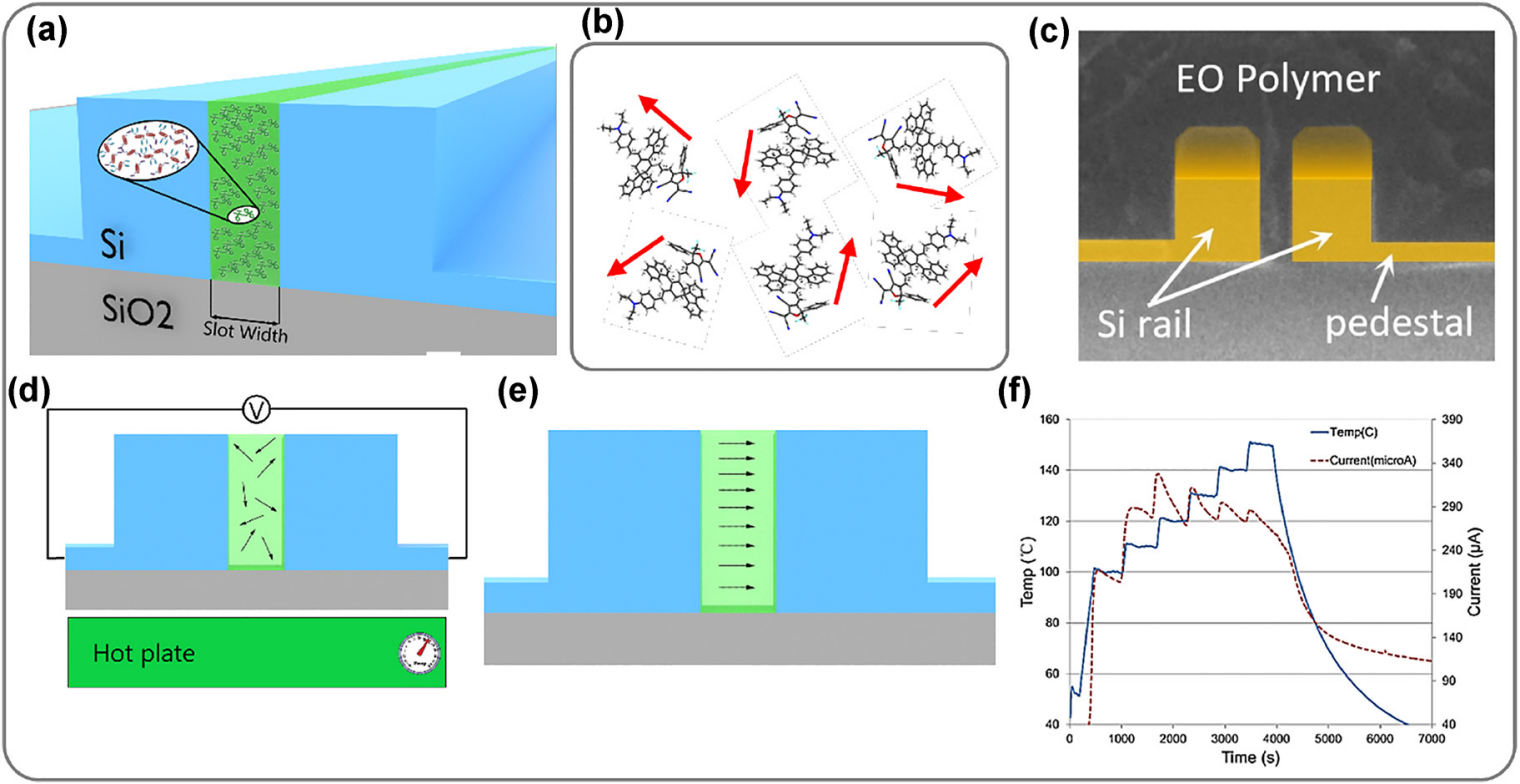
(a) Cross-section of a slot-waveguide infiltrated with EOP polymer. (b) Chromophore dipoles in polymer host. Dipoles are not aligned before poling [54]. (c) False-colored SEM image of the slot indicating almost-perfect infiltration of SEO125 polymer [97]. (d) Electrothermal poling set-up: under applied external (DC) field, the sample is heated at a determined rate (typically 10 °C/min) up to 95 °C and then stabilized for 10 additional minutes for each 10 °C of temperature increase. This is somewhat crucial to allow dipole orientation. Finally, the EOP sample reaches the glass temperature (*T*_g_). (e) While maintaining the poling field, the sample is cooled down to room temperature as rapidly as possible followed by removing the poling field. (f) Temperature and leakage current should be monitored during the poling procedure. Figure reproduced with permission from  [32], ACS.

As illustrated in Figure 4, the electrothermal poling process can result in a leakage current through the EOP. The relationship between the electrical conduction phenomena and the high-electric-field poling of a chain-polymer has been investigated in [96]. At poling fields >100 V/*μ*m, the leakage is dominated by the tunneling current due to the Fowler-Nordheim effect. Leakage currents are detrimental to the poling efficiency. They should be prohibited by adding an additional layer of charge barrier such as bisbenzcyclobutene (BCB), high-*κ* dielectric metal oxide such as TiO_2_ [97, 98] or Al_2_O_3_ [99], HfO_2_ [82] or poly-methyl-siloxane layer [100] between the polymer and the electrode. An alternative to reduce leakage and improve the poling efficiency (by decreasing the required voltage to establish the poling field) is to use a TiO_2_-modified transparent electrode, with which an *r*_33_ of 350 pm/V has been demonstrated in [101]. A graphene electrode is also proposed in [102] but the complexity of adding graphene makes this technique challenging. Surface passivation using high-*κ* dielectric metal oxide can further improve infiltration (by making hydrophilic sidewalls) [97] and decrease pedestal resistance [103], thereby improving speed. Almost perfect infiltration for narrow slots has been reported in [97, 99]. The resistive slab layer (i.e., the pedestal) can be replaced with high-*κ* (e. g., BaTiO_3_) slotlines to build a capacitively coupled SOH structure [104], whose BW is not limited by the RC time constant of the slot. Additionally, lower IL can be achieved since Si rails do not need to be doped in this structure. This MZM combines the benefits of a slot waveguide (i.e., low *V*_*π*_ *L*) and a non-slotted one (i.e., lower IL and higher BW). The benefits of this architecture lie between POH and SOH.

## 4 Challenges, future roadmap, and conclusion

EOP modulators can be used in applications from telecommunication and data communication [83, 105–108] to optical interconnects [49, 109] and RF sensing [110, 111]. Despite their several advantages, the high-volume production of polymer-based modulators remains a challenge. Although significant improvements in the *T*_g_ of the EOP have been made (Table 2), the limited *T*_g_ still poses a challenge with the existing SiP manufacturing process that requires exposure to a higher temperature. Beyond *T*_g_, EOPs lose their electro-optical properties. One workaround is to shift the poling step to the last step of fabrication to ensure that the EOP remains intact during other process steps. However, the need for poling and executing it as the final step of manufacturing challenges to the existing fabrication flow that must be resolved for high-volume production. Poling of narrow slot waveguides can be challenging too. As discussed earlier, it requires extra surface passivation steps to avoid poor poling efficiency.

**Figure 5:**
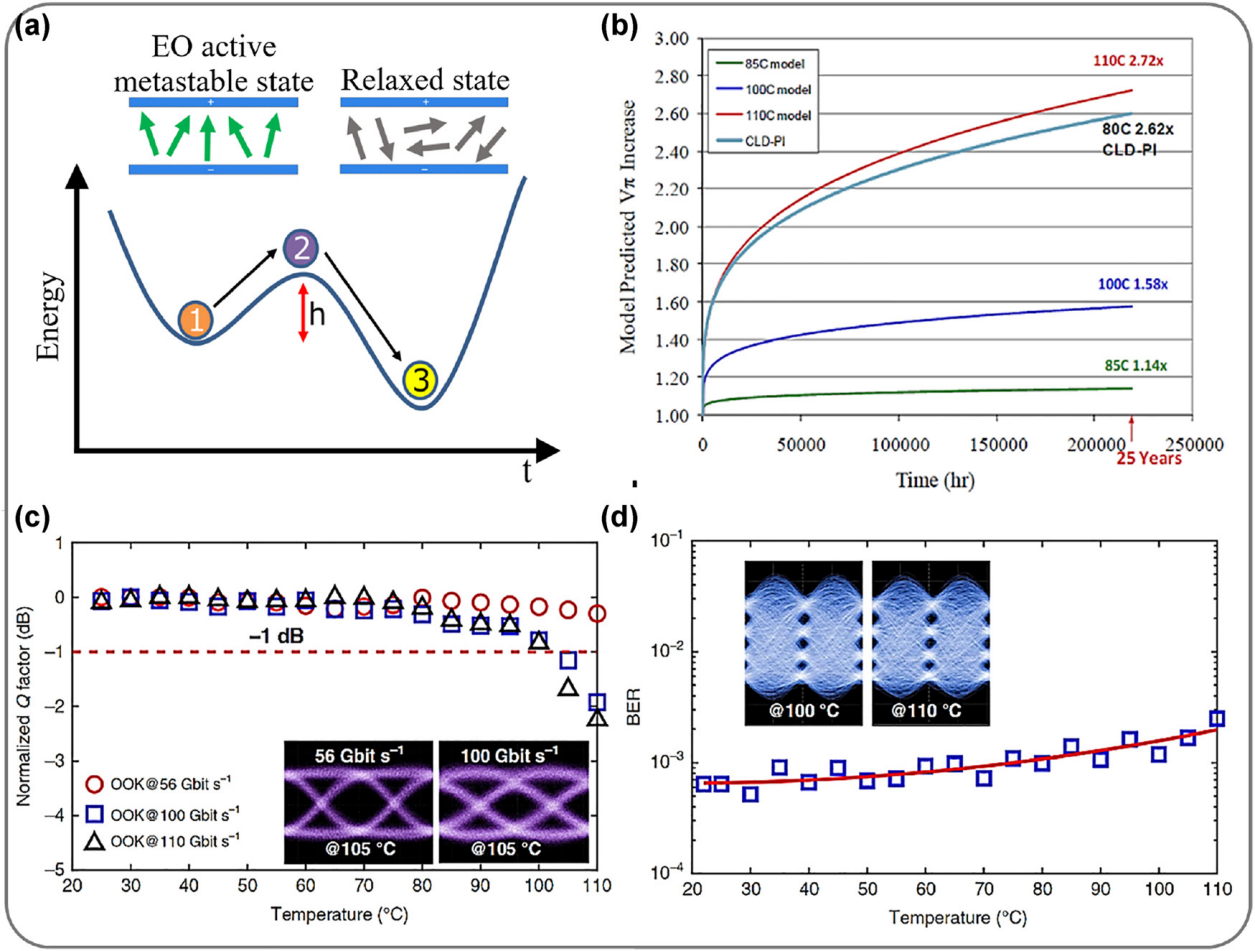
(a) Poled polymers transition from their metastable (“1”) state to the relaxed state (“3”) at a rate controlled by the barrier height (*h*) [54]. (b) Extrapolation of normalized *V*_*π*_ increase for two different EOP modulators for various temperatures. Figure reproduced with permission from [112], SPIE. (c) Normalized *Q* factors (dB) for OOK at 56, 100 and 110 Gb/s versus operating ambient temperature (25–110 °C). Inset: measured optical eye diagrams of 56 and 100 Gb/s at elevated temperature (105 °C) [37]. (d) Measured BERs of 200 Gb/s PAM4 versus operating ambient temperatures (22–110 °C). The solid red line represents the fitted curve. Inset: measured optical eye diagrams of 200 Gb/s PAM4 at elevated temperatures (100–110 °C) [37]. Distributed under Creative Commons license CC BY 4.0.

Another challenge for organic material is aging and long-term reliability. Figure 5a shows the energy state diagram of the poled polymer [54]. State 1 shows the EO metastable state where the molecular dipoles are still aligned. The polymer will slowly transition to the relaxed state 3. The higher the barrier height (*h*), the slower this process would occur. Surrounding the chromophore dipoles with more host polymers, and modifying the chromophores to act as anti-plasticizers, and increasing the rigidity are two techniques to increase *h* [54]. Cross-linkable binary molecular polymers also promise long-term stability [37]. Figure 5b shows the model-predicted *V*_*π*_ increase over time at various operating temperatures for an EOP modulator described in [112], suggesting 25 years of reliable operation at 85 ^o^C. Researchers in [113] demonstrate the operation of EOP coated MZM modulators with thermophysical stable polymers which can withstand up to 110 ^o^C. Other temporal stability measurements accomplished in [32, 114] suggest no degradation in *r*_33_ up to 2000 h at 85 ^o^C. EOP modulators have been shown to pass reliability testing (standards GR-468) where low and high temperatures, vibration and mechanical shock, fiber twist, and thermal shock are investigated [115]. Figure 5c and d show the operational stability of the EOP modulators as a function of temperature, represented by the normalized *Q*-factor for on-off keying (OOK) and BER for PAM4 signaling [37]. Finally, long-term operation of SOH modulators must be demonstrated without hermetically sealed packages or lower-cost packaging must be developed for hermeticity [116]. A recent study [114] suggests that moderate barriers against water vapor transmission may prove adequate without the need for full hermetic enclosures.

As evident from Table 1, SOH modulators have about 10× better modulation efficiency and loss efficiency, and about 2× higher BW than their FCD counterparts. Nevertheless, a significant performance gap exists between the SOH and POH modulators in terms of *V*_*π*_ *L* and BW, serving as a motivation to improve the SOH modulators further. Improving of the polymer’s electro-optic and physical/chemical properties will help considerably. Increasing the *r*_33_ will lower the *V*_*π*_ *L* for the SOH modulators further. Since polymer modulators are typically limited in BW due to the RC effect, a lower *V*_*π*_ *L* can lead to smaller devices and even larger BW. Improvement in the design of the device and the EOP material loss can further reduce the IL [83].

Silicon photonic provides low-cost photonic chips in high-volume production, in millions of quantities. Existing research and development in free-carrier plasma dispersion modulators in silicon face challenges in *α* × *V*_*π*_ *L* reduction and improving bandwidth. The addition of Ge, InP, or polymer materials to the CMOS silicon foundry seems promising to usher the next generation of high-volume high-performance modulators. SOH modulators have demonstrated convincing performance already, and further demonstrations of higher *r*_33_, aging, reliability, manufacturing, and packaging will lead to its adoption into the next generation of silicon photonics.
